# Increased Proinflammatory Cytokine Levels in Prolonged Arthralgia in Ross River Virus Infection 

**DOI:** 10.3201/eid2304.160466

**Published:** 2017-04

**Authors:** Dennis Tappe, José Vicente Pérez-Girón, Sergio Gómez-Medina, Stephan Günther, César Muñoz-Fontela, Jonas Schmidt-Chanasit

**Affiliations:** Bernhard Nocht Institute for Tropical Medicine, Hamburg, Germany (D. Tappe, S. Günther, C. Muñoz-Fontela, J. Schmidt-Chanasit);; German Centre for Infection Research, Hamburg (D. Tappe, S. Günther, C. Muñoz-Fontela, J. Schmidt-Chanasit);; Leibniz Institute for Experimental Virology, Hamburg (J.V. Pérez-Girón, S. Gómez-Medina, C. Muñoz-Fontela)

**Keywords:** arthralgia, arthritis, viruses, cytokines, Ross River virus, Australia, mosquitoes, vector-borne infections

## Abstract

Ross River virus, a mosquitoborne alphavirus, causes epidemic polyarthritis in Australia and the Pacific region. We analyzed serum cytokine, chemokine, and growth factor levels in travelers returning to Germany from Australia. Serum samples showed elevated concentrations in the acute phase of the illness and, more pronounced, in the long-lasting convalescent phase.

Ross River virus (RRV) is a mosquitoborne alphavirus endemic to Australia and the Pacific region. The virus is transmitted by various *Aedes* and *Culex* spp. mosquito species; macropods are the natural reservoir (*1*). RRV causes epidemic polyarthritis, with several thousand cases noted annually in Australia (*1*). Large outbreaks occurred during 1979–1980 on South Pacific islands. Infections in travelers, however, are rare (*2*). Epidemic polyarthritis is a self-limiting febrile arthralgia syndrome that closely resembles chikungunya and Mayaro fever. Acute-onset symmetric polyarthritis, most often affecting the fingers, wrists, ankles, and knees, is the predominant clinical presentation. Rash, myalgia, fatigue, and fever are present in half of patients (*1*). Joint effusions are common, and arthralgia can be long-lasting (months to years), and recurring. 

The clinical disease, diagnostic procedures, and epidemiology of RRV have been well described, but immunologic parameters in humans and their usefulness in the clinical follow-up of patients warrant further investigation. Therefore, we investigated travelers returning to Germany from Australia with epidemic polyarthritis resulting from RRV infection.

We analyzed 20 serum samples from 16 patients (7 men, 9 women; age range 20–67 years [median 38 years]) who had serologic evidence for acute or recent RRV infection, as confirmed by indirect immunofluorescence assay and virus neutralization assay (*2*) (Table). All patients had acquired the infection during travel in Australia. We obtained serum samples at different times after symptom onset (3 days–16 weeks) and classified them as either acute (taken <30 days after symptom onset; n = 7) or convalescent with symptomatic arthralgia (taken >30 days after disease onset; n = 13). After we obtained written consent from the patients, we subjected the serum samples to multiplex cytokine analyses. In parallel with the RRV patients, we tested 20 serum samples from healthy blood donors. 

**Table:** Returning travelers from Australia infected with Ross River virus included in study of cytokine levels during acute and convalescent disease phases, Germany

Patient no.	Age, y/sex	Timing of cytokine level testing after symptom onset	IgM titer*	IgG titer*
1	33/M	8 wk	1:320	1:320
2	67/F	7 wk	1:80	1:1,280
3	55/F	4 wk	1:2,560	1:1,280
4	24/M	10 and 13 wk	1:320 each	1:2,560
5	29/F	6 wk	1:2560	1:10,240
6	21/F	6 wk	1:80	1:1,280
7	46/M	5 d	1:2,560	1:80
8	31/F	1 wk	1:10,240	1:160
9	32/F	2 wk	1:81,900	1:327,680
10	51/F	4 and 8 wk	1:320 and 1:160	1:2,560 and 1:640
11	20/F	6 wk	1:320	1:2,560
12	47/M	8 wk	1:160	1:320
13	32/M	16 wk	1:20	1:160
14	43/F	8 wk	1:320	1:2,560
15	53/M	3 d, 2 wk, and 9 wk	1:320, 1:10,240, and 1:640	1:20, 1:1,280, and 1:320
16	49/F	6 wk	1:1,280	1:2,560

*By indirect immunofluorescence testing; reference value <1:20 (*2*).

Compared with concentrations in samples from healthy controls, concentrations in samples from patients in the acute phase of RRV infection showed noticeably elevated concentrations of serum interleukin (IL) 4 and 7; granulocyte-macrophage colony stimulating factor (GM-CSF); regulated on activation, normal T cell expressed and secreted (RANTES); interferon-γ–induced protein 10 (IP-10); and vascular endothelial growth factor (VEGF). We also saw a notable decrease in eotaxin levels in acute-phase samples. In samples from patients in the arthralgic convalescent phase, we noted increases in IL-1β, IL-4, IL-6, IL-8, IL-9, IL-13, IL-15, GM-CSF, interferon-γ, tumor necrosis factor-α (TNF-α), RANTES, basic fibroblast growth factor (bFGF), macrophage inflammatory protein 1α (MIP1α), and VEGF in comparison to healthy controls. Substantial elevations in the convalescent phase when compared with the acute phase were recorded for levels of bFGF and MIP1α, and a marked decrease was seen in IP-10 concentrations.

Cytokine and chemokine levels were generally higher in the convalescent phase than in the acute phase, with individual exceptions (Technical Appendix). No notable changes in either phase were seen for IL-1RA, IL-2, IL-5, IL-10, IL-12p70, IL-17, granulocyte colony-stimulating factor, monocyte chemotactic protein 1, and platelet-derived growth factor β polypeptide levels (data not shown), and macrophage inflammatory protein 1β concentrations.

Similar to chikungunya and Mayaro fever, the most prominent clinical symptom of RRV infection is long-lasting arthralgia. Nearly 60% of patients reported persisting pain after 2–3 years (*1*). RRV RNA has been detected in synovial fluid up to 5 weeks after symptom onset (*3*), suggesting ongoing viral replication and inflammation (*4*). In the RRV-infected travelers examined in this study, the increased proinflammatory cytokine serum concentrations during the prolonged arthralgic convalescence phase strengthen the hypothesis of persisting inflammation of the joints. In patients and in a murine model, macrophage-derived TNF-α, interferon-γ, and IL-6 were elevated in synovial fluid during RRV disease (*5*), as we have shown here in serum.

Furthermore, increased levels of IL-1β, IL-6, IL-15, MIP1α, and GM-CSF, as seen in our study, have been described in the clinically similar chikungunya (*6*). Elevated levels of RANTES (and IP-10 initially) indicate T-cell activation, possibly reflecting ongoing viral replication in the joints, also as described in chikungunya (*6*). A similar effect could recently be demonstrated in Mayaro fever patients with prolonged arthralgia (*7*).

In conclusion, cytokine level testing in alphavirus infections with prolonged arthralgia may aid monitoring patient symptoms. This information is particularly valuable when clinical signs of arthritis, such as joint swelling and redness, are no longer present, and standard serum inflammatory parameters are within reference ranges. Low-grade inflammation in persistent alphavirus-induced arthritis might place patients at risk for bone loss and fractures (*8*). Levels of RANKL (receptor activator of nuclear factor κB ligand), which were not determined in our study, were recently shown to be elevated in RRV infection and associated with increased osteoclast formation (*9*).

The pattern of cytokine concentration elevations we demonstrated for patients with epidemic polyarthritis is similar to what has recently been described for patients with Mayaro fever (*7*) and chikungunya (*6*,*10*). Our data broaden the knowledge of alphavirus pathogenesis in arthralgia syndromes; however, more immunological investigations, including human T-cell analyses, are needed.

Technical Appendix Changes in cytokine, chemokine, and growth factor levels in the acute and prolonged arthralgia (convalescent) phases of Ross River virus infection, along with data on 2 sample patients.
