# Supplementary material for: Increased Proinflammatory Cytokine Levels in Prolonged Arthralgia in Ross River Virus Infection
Source: Emerg Infect Dis. 2017 Apr;23(4):702–4. doi: 10.3201/eid2304.160466 (PMC5367403; doi:10.3201/eid2304.160466)
Supplement: Technical Appendix — Changes in cytokine, chemokine, and growth factor levels in the acute and prolonged arthralgia (convalescent) phases of Ross River virus infection, along with data on 2 sample patients. [file 16-0466-Techapp-s1.pdf]

Article DOI: http://dx.doi.org/10.3201/eid2304.160466

Increased Proinflammatory Cytokine Levels in Prolonged Arthralgia in Ross River Virus Infection

Technical Appendix

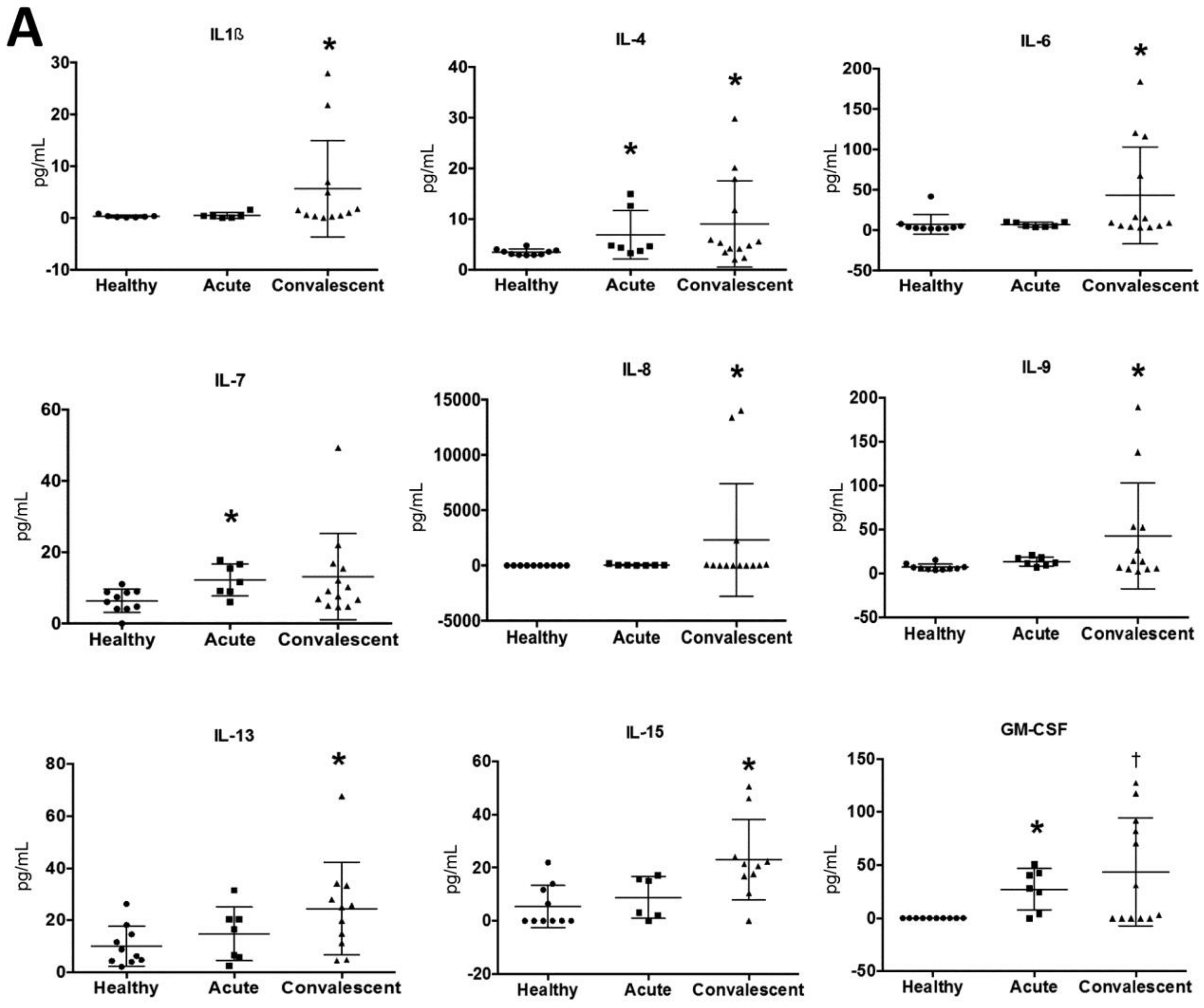

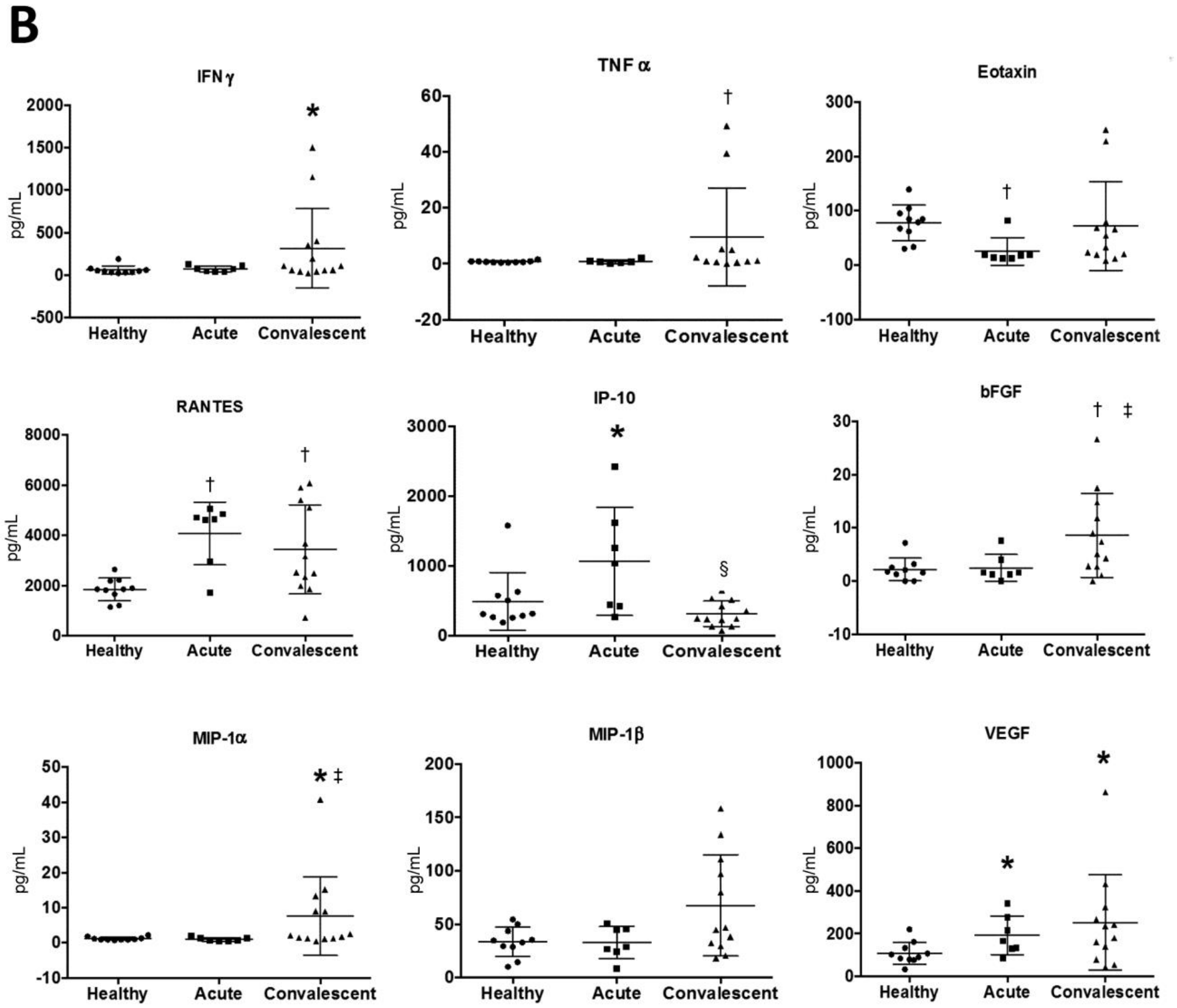

Technical Appendix Figure 1. Changes in cytokine, chemokine, and growth factor levels in the acute and prolonged arthralgia (convalescent) phase of Ross River virus infection. A) Serum levels of IL-4, IL-7 and GM-CSF were elevated during the acute phase of the infection, whereas IL-1β, IL-4, IL-6, IL-8, IL-9, IL-13, IL-15, and GM-CSF demonstrated notable increases during the prolonged arthralgic convalescent phase when compared with healthy controls. B) Increased serum concentrations of RANTES, IP-10, and VEGF during the acute phase, with decreased eotaxin levels at that time. Concentrations of IFN-γ, TNF-α, RANTES, bFGF, MIP1α (but not MIP1β), and VEGF were elevated in the arthralgic convalescent phase when compared with healthy controls. IP-10, bFGF, and MIP1α concentrations also showed changes in the convalescent phase when compared with the acute phase. *$p<0.05$, †$p<0.01$, acute-phase or convalescent-phase serum versus healthy controls; ‡$p<0.05$, §$p<0.01$ convalescent-phase versus acute-phase serum (by Kruskal-Wallis test). bFGF, basic fibroblast growth factor; IFN, interferon; IP, interferon-γ-induced protein; MIP, macrophage inflammatory protein; RANTES, regulated on activation, normal T cell expressed and secreted; TNF, tumor necrosis factor; VEGF, vascular endothelial growth factor.

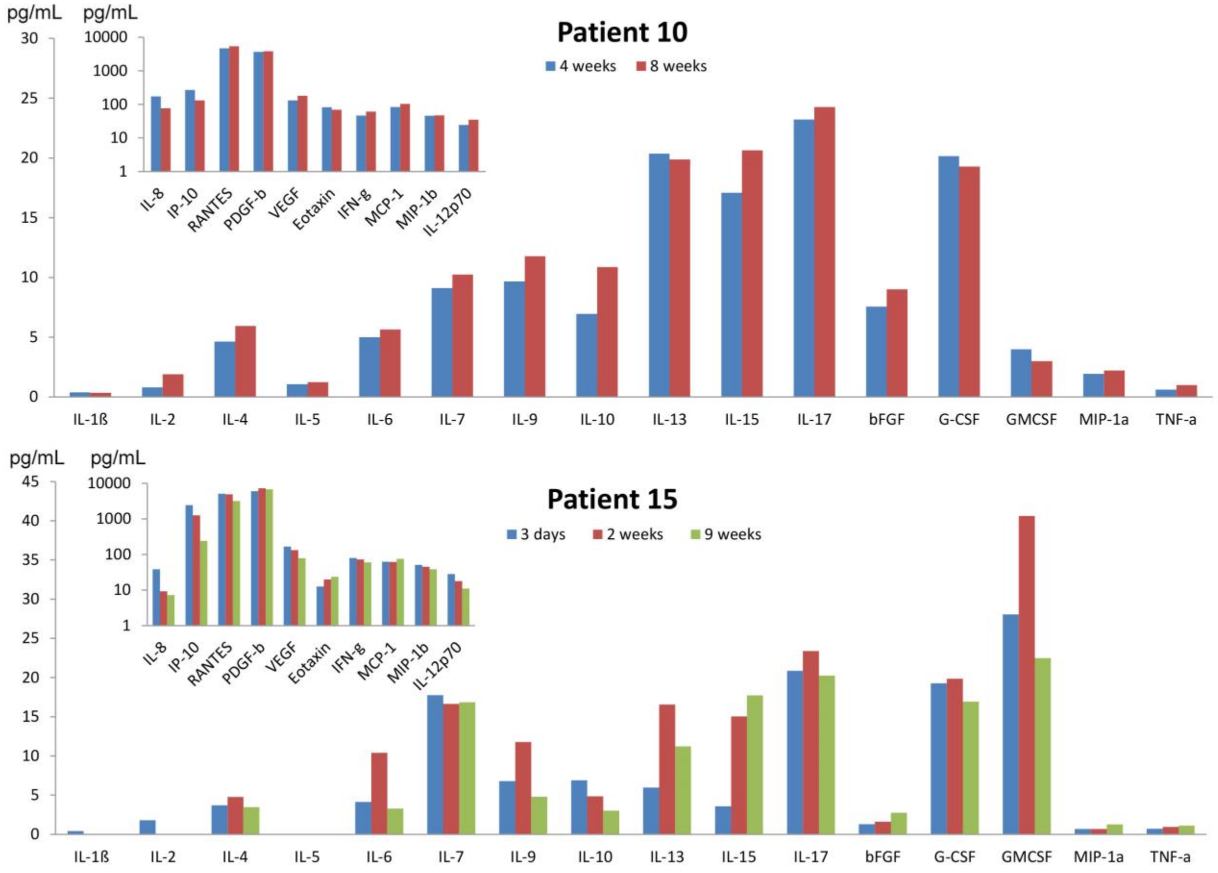

Technical Appendix Figure 2. Serum cytokine, chemokine, and growth factor level changes in 2 individual patients over time. In the 2 patients depicted, several parameters were lower in the convalescent phase than in the acute phase.
